# Methodological approach to create an atlas using a commercial auto‐contouring software

**DOI:** 10.1002/acm2.13093

**Published:** 2020-11-25

**Authors:** Marta Casati, Stefano Piffer, Silvia Calusi, Livia Marrazzo, Gabriele Simontacchi, Vanessa Di Cataldo, Daniela Greto, Isacco Desideri, Marco Vernaleone, Giulio Francolini, Lorenzo Livi, Stefania Pallotta

**Affiliations:** ^1^ Department of Medical Physics Careggi University Hospital Florence Italy; ^2^ Department of Experimental and Clinical Biomedical Sciences University of Florence Florence Italy; ^3^ National Institute of Nuclear Physics (INFN) Florence Italy; ^4^ Department of Radiation Oncology Careggi University Hospital Florence Italy; ^5^ Florentine Institute of Care and Assistance (IFCA) Florence Italy

**Keywords:** automatic contouring, atlas, CT, methodological approach, pelvis, radiotherapy, segmentation

## Abstract

**Purpose:**

The aim of this work was to establish a methodological approach for creation and optimization of an atlas for auto‐contouring, using the commercial software MIM MAESTRO (MIM Software Inc. Cleveland OH).

**Methods:**

A computed tomography (CT) male pelvis atlas was created and optimized to evaluate how different tools and options impact on the accuracy of automatic segmentation. Pelvic lymph nodes (PLN), rectum, bladder, and femurs of 55 subjects were reviewed for consistency by a senior consultant radiation oncologist with 15 yr of experience. Several atlas and workflow options were tuned to optimize the accuracy of auto‐contours. The deformable image registration (DIR), the finalization method, the k number of atlas best matching subjects, and several post‐processing options were studied. To test our atlas performances, automatic and reference manual contours of 20 test subjects were statistically compared based on dice similarity coefficient (DSC) and mean distance to agreement (MDA) indices. The effect of field of view (FOV) reduction on auto‐contouring time was also investigated.

**Results:**

With the optimized atlas and workflow, DSC and MDA median values of bladder, rectum, PLN, and femurs were 0.91 and 1.6 mm, 0.85 and 1.6 mm, 0.85 and 1.8 mm, and 0.96 and 0.5 mm, respectively. Auto‐contouring time was more than halved by strictly cropping the FOV of the subject to be contoured to the pelvic region.

**Conclusion:**

A statistically significant improvement of auto‐contours accuracy was obtained using our atlas and optimized workflow instead of the MIM Software pelvic atlas.

## 1 INTRODUCTION

In radiotherapy planning, image segmentation is one of the preliminary and time‐consuming tasks, affected by interobserver variability.^1^, ^2^, ^3^, ^4^, ^5^, ^6^ This procedure, usually performed on computed tomography (CT) images, is affected by the scarce image contrast that hinders the application of semi‐automatic segmentation algorithms based on threshold or region growing. Moreover, some of the criteria to define targets and organs at risk (OAR) are not related to CT visible anatomical boundaries. For these reasons, image segmentation for radiotherapy treatment planning is still a challenging and labor‐intensive task. Semi‐automatic contouring methods are implemented in commercial treatment planning systems (TPS) to support users in reducing contouring time, but only atlas‐based or artificial intelligence methods can aim to fully automate the contouring processes.

The atlas‐based approach relies on the availability of one or more CT series of a certain anatomical district, already contoured by an expert physician following guidelines.^7^, ^8^ The operating principle is to perform a deformable registration of an atlas subject on the new subject and then apply the same transformation to the atlas structures, to obtain a proposal of contouring for the new subject. While the single‐subject approach uses only one subject, the multisubject one uses N subjects, which should better represent patients' anatomical variability. In principle, each subject of the multisubject atlas could be deformed on the subject to be contoured, to obtain N possible sets of structures. To save time and to increase the segmentation accuracy, a reduced set of k subjects, the most similar to the patient to be contoured, can be used. From each of the k subjects, a contours proposal is derived, and finally, a finalization algorithm combines these k series of contours into a single set of contours.

Although contours obtained with atlas‐based algorithms require some minor or major editing,^9^ the atlas approach has been proved to be effective in reducing CT contouring time and interoperator variability for various anatomical sites.^2^, ^17^

In the last years, the increasing availability of computing power and storage space has promoted the development of automatic segmentation methods based on artificial intelligence and machine learning approaches.^3^, ^18^, ^19^, ^20^, ^21^ Some of these methods have been proved to be very effective to produce accurate contours requiring minimal editing by physicians,^20^ but their implementation and training is very demanding. Even when neural networks are implemented in commercial software, hospitals usually do not have the possibility to collect an adequate training set of studies.^21^

For these reasons, atlas‐based segmentation remains a reasonable option for automated contouring in radiotherapy and it is implemented by several vendors, both as a TPS option or as a stand‐alone software module (Table S1).

Several studies about CT images automatic segmentation of various anatomical sites have been published,^2^, ^27^ but only few of them^24^, ^27^ reported in detail the methodology adopted for atlas creation. Most published works briefly described the used atlas and then investigated the effect of automated contouring introduction into clinical workflow in terms of time sparing^2^, ^5^, ^10^, ^11^, ^12^, ^13^, ^14^, ^15^, ^16^ and interobserver variability.^2^, ^5^, ^14^, ^17^

Thus, the aim of this work was to focus on the methodology for atlas generation and on a workflow for automatic contouring using MIM MAESTRO (MIM Software Inc., Cleveland, OH) software. A CT male pelvis atlas was created and optimized to evaluate how different tools and options impact on the accuracy of automatic segmentation. The methodology presented here permits to understand strength and weakness of each tool, besides learning how to take full advantage of MIM MAESTRO automatic contouring tools. We believe that this step‐by‐step analysis might guide the creation and optimization of atlases and workflows for automatic segmentation of any anatomical sites.

## 2 MATERIALS AND METHODS

MIM MAESTRO v.6.8.2 (MIM Software Inc., Cleveland, OH), installed on a workstation with Intel Core i7‐4770 CPU and 16 GB RAM, was used to create a CT atlas of male pelvis. This software adopts a multisubject atlas‐based segmentation method that enables users to select both atlas subjects and atlas representative subject. Moreover, MIM MAESTRO software offers the possibility to embed the atlas into a customizable workflow which allows users to set several options, such as registration algorithm and finalization method, and to implement some post‐processing operations.

In order to manage the large number of possible combinations of atlas and workflow parameters, a two‐step process was employed. In the first step, the best atlas was identified by using a standard workflow, while in the second step, the influence of all the workflow parameters was investigated using the best atlas version previously selected.

### 2.A Atlas

To create an atlas, it is necessary to select some subjects and to register each of them on a reference subject. This reference subject, also named template, is chosen as atlas representative subject. During atlas construction, each subject is registered on the template, using a rigid algorithm, to determine a similarity index, which aims to quantify the anatomical affinity of each atlas subject to the template. When the atlas is used to segment a new patient dataset, the patient's CT study is registered on the atlas template and a similarity index is evaluated. This value is compared to the similarity indices of all the atlas subjects in order to choose the subject, or the k subjects in a multisubject approach, which best matches the patient anatomy.

#### 2.A.1 Atlas creation

##### Subjects and template selection

Fifty‐five CT male pelvis studies were used to build the atlas. A Brilliance BigBore CT scanner (PHILIPS Healthcare) was used to acquire CT studies (120 kV, 600 mm FOV, 512 × 512 matrix, and 3 mm slice thickness). Data were selected among CT images of patients treated for prostate cancer, with intact prostate and no known nodal involvement. Computed tomography studies of patients with prosthesis, calcifications and other high‐density elements, or acquired with contrast medium were excluded.

**Fig. 1:**
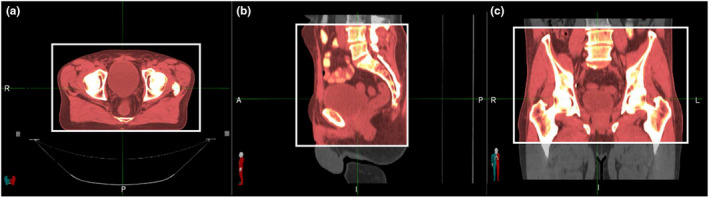
Criteria used for field of view cropping. Axial, sagittal, and coronal views are showed, from left to right. The crop extension is represented by the white box.

As the field of view (FOV) and longitudinal extension of CT studies widely exceeded the pelvic area, all datasets were preprocessed. The FOV was manually cropped to exclude the CT couch‐top posteriorly and to include the external patient contour plus about 1 cm of air in the other directions. In the superior/inferior direction, CT studies were limited to L3/L4 edge and to include the lesser trochanter of the femur (Fig. 1).

Original manual contours of pelvic lymph nodes (PLN), rectum, bladder, and femurs, used for treatment planning, were reviewed for consistency by a senior consultant radiation oncologist with 15 yr of experience, according to RTOG criteria and Taylor et al. guidelines^28^ for PLN, before adding them to the atlas. Prostate was not contoured as previous studies,^26^, ^29^, ^30^ demonstrated that atlas based on CT image segmentation resulted in suboptimal prostate contours. In case of prostate, better results can be achieved using atlases based on MRI images,^31^ or using deep learning.^3^, ^19^, ^32^

The choice of the atlas template subject was performed based on a semiobjective criterion, according to the following four classes: patient and bladder size, and bladder position and shape. For each class, the most representative subject was chosen, and among them, the typical subject, able to resume all the characteristics, was selected to represent the atlas template.

##### Registration of the subjects on the template

During the automatic registration of each atlas’ subject on the template, the operator can decide whether making manual corrections or not. To investigate if this operation might influence automatic segmentation accuracy, two atlases, with the same template and the same subjects, were created. In the first case (Atlas 1), the registration of each subject on the model was performed roughly aligning all the pelvis structures, while in the second case (Atlas 2), this was executed trying to align primarily the bladder, as it turned out to be the most challenging structure for the tested auto‐contouring algorithm, presumably due to the huge anatomical variability.

In order to test the influence of FOV and longitudinal extension of CT studies on automated‐contouring time, another atlas (Atlas 3) was created using the original 55 CT studies without image cropping. Each atlas subject was registered on the template aligning the bladder (as in Atlas 2).

#### 2.A.2 Atlas performances

Atlas 1 and Atlas 2 performances were evaluated on a subset of 20 subjects, randomly selected from those used to create the atlas, with a leave‐one‐out approach, that is using a software option to exclude the subject from the atlas while performing its own segmentation. Automatic contours, obtained with each atlas version, were compared to the reference ones (manually contoured by the radiation oncologist), and similarity indices (see Section 2.D) were evaluated for each patient and region of interest (ROI).

Atlas 2 and Atlas 3 were used to generate the contours of four test subjects. Two versions of the same subject (CT or CT cropped) were used, thus resulting in four configurations: Atlas 2 + CT, Atlas 2 + CT cropped, Atlas 3 + CT, Atlas 3 + CT cropped. Contouring times were registered and compared.

### 2.B Workflow customization and optimization

In the used workflow, for the atlas invoking, it is possible to select and customize the following settings: the deformable registration method, the finalization algorithm, and the number of subjects used by the multisubject atlas. In order to regularize any odd shape of the contours,^9^ it is convenient to add some postprocessing functions to the workflow. In our case, we used the following tools: smooth, clean, fill holes, and Hounsfield Unit (HU) range lock. After a rough examination of ROI HU histograms for some test patients, we adopted the following ranges: bladder (−39, 37), rectum (−60, 80), pelvic nodes (−175, 100), femurs (>150).

#### 2.B.1 Deformable image registration (DIR) algorithm

The core of any atlas‐based contouring software is the deformable registration algorithm. MIM MAESTRO software is equipped with *Same‐subject* and *Multi‐modality* algorithms (Piper JW, Richmond JH, Nelson AS. VoxAlign Deformation Engine ® Deformable Algorithms; 2018)^33^.

The first is the standard method for mono‐modal registration, while the latter is suitable for multi‐modality images or in those cases when the standard algorithm fails.

The DIR deformation can be tuned choosing a smoothing factor (SF), variable in the range 0 to 1. The lower the SF, the higher the degree of deformation allowed. Automatic contours, obtained using *Same‐subject* algorithm and three SF values (0.1, 0.5, 1), were compared to the 20 test patients reference contours and similarity indices were evaluated.

From this analysis, the best SF factor was assessed and then used to compare the *Same‐subject* and *Multi‐modality* algorithms. The most accurate DIR algorithm was included in the customized workflow.

##### Automatic reg refine option

The software provides also an automatic option to refine the deformable registration: *Automatic Reg Refine* (*ARR*). When this option is activated, the software automatically creates a set of bounding boxes, with central point evenly spaced on the surface of atlas subject contour. Inside these boxes, a local rigid algorithm is used to maximize the match between reference (patient to be contoured) and deformed (atlas subject) studies. We tested the following three configurations: *ARR* inactivated (A), *ARR* activated with default options, that is, 60 mm box spacing and 30 mm box size (B), *ARR* activated with modified options, that is, 30 mm box spacing and 20 mm box size (C). The three options were tested on a test sample of 20 patients.

#### 2.B.2 Finalization algorithm

Majority Vote (MV) and STAPLE^34^ are the finalization methods available to create, from the k set of contours, a single set. In case of MV, a voxel is assigned to a certain structure if that voxel belongs to the same structure for most k subjects. STAPLE, which is based on an expectation–maximization algorithm, considers the collection of k segmentations and computes a probabilistic estimate of the true segmentation. Automatic contours obtained with the two finalization methods were compared to the reference contours of the 20 test patients and similarity indices were evaluated. The finalization algorithm providing better results was included in the customized workflow.

##### Finalization algorithm: # of k best matching subjects

In the multisubject atlas, a selectable number of subjects (k) can be used to extract multiple sets of contours. The multisubject approach has been proved to be more effective than the single‐subject one.^24^, ^35^ Nonetheless, the selection of a reduced set of best matching subjects could, in principle, reduce auto‐contouring time and increase accuracy of the generated contours.^27^, ^35^

Different numbers of best matching subjects (k) were tested (5, 9, 13, 17, 21). Automatic contours generated for each k value were compared to the reference contours of the 20 test patients and similarity indices were evaluated. The k value maximizing the accuracy of all ROIs was included in the customized workflow.

### 2.C Customized atlas and workflow vs MIM provided atlas and workflow

MIM Software is provided with proprietary atlases (not editable) and predefined editable workflows. A proprietary atlas contains a set of anonymized CT series, with contours vetted before distribution (MIM atlas high‐risk prostate contains 38 subjects).

To assess how the fine‐tuning of the workflow parameters and the use of a locally developed atlas might impact on the contours’ accuracy, we extracted the automatic contours of the same 20 test patients used in this study for other tests, using the following three different configurations: MIM 6.9.5 default atlas and workflow, MIM atlas and our customized workflow, our atlas and customized workflow. For the first option (MIM default atlas and workflow), we decided to use MIM software version 6.9.5 (which became available only at the end of the work), considering that most readers will use 6.9.5 or later versions. Automatic contours obtained with these three approaches were finally compared to the reference contours and similarity indices were evaluated.

### 2.D Similarity indices and statistical analysis

#### 2.D.1 Similarity indices

To quantify the accuracy of atlas‐based contours, several parameters can be used to compare them to reference contours. MIM MAESTRO software provides a tool which automatically calculates Jaccard similarity coefficient (JSC), Dice similarity coefficient (DSC), mean distance to agreement (MDA), and Hausdorff distance (HD) between two contours. JSC and DSC indices are both an expression of contours spatial overlapping, while MDA and HD quantify the average and maximum distance between contours. Similar contours will exhibit DSC and JSC values near 1 and low MDA and HD values. In order to reduce redundancy, we selected just two indices: DSC, as it is more widespread in the literature; and MDA, which is better than HD in highlighting the contours that need a more demanding contour editing in the refinement phase. A regularly shaped contour, characterized by many regions with small deviations from the reference contour, presents a higher MDA while a contour which differs from the reference for a single spike is better evidenced by HD. The latter case is obviously easier to correct.

#### 2.D.2 Statistical analysis

For each ROI, similarity indices for each tested parameter were statistically compared to test differences significance. To guide the choice for the appropriated statistical test, between parametric and nonparametric ones, a normality test of Shapiro–Wilk was conducted. For two groups, *t*‐test and Wilcoxon signed rank test were used for normally and not‐normally distributed data, respectively. For multiple groups (par. 2.2.2, k optimization), ANOVA and Friedman test for normally and not normally distributed data, respectively, were performed. Regardless of the chosen statistical test, two‐tailed analyses were always performed and a significance level of 0.05 was adopted. Online calculators were used to perform statistical tests: Shapiro–Wilk test,^36^, ^37^ ANOVA,^38^ Friedman,^38^ paired *t*‐test,^38^ and Wilcoxon signed rank test.^38^

The choice of each parameter was guided by the output of the statistical test, as follows: in case of significant difference, the best option was selected; in case the statistical test did not highlight any significant difference, we maintained the default option.

## 3 RESULTS

### 3.A Atlases performances

#### 3.A.1 Accuracy of auto‐contours

**Fig. 2:**
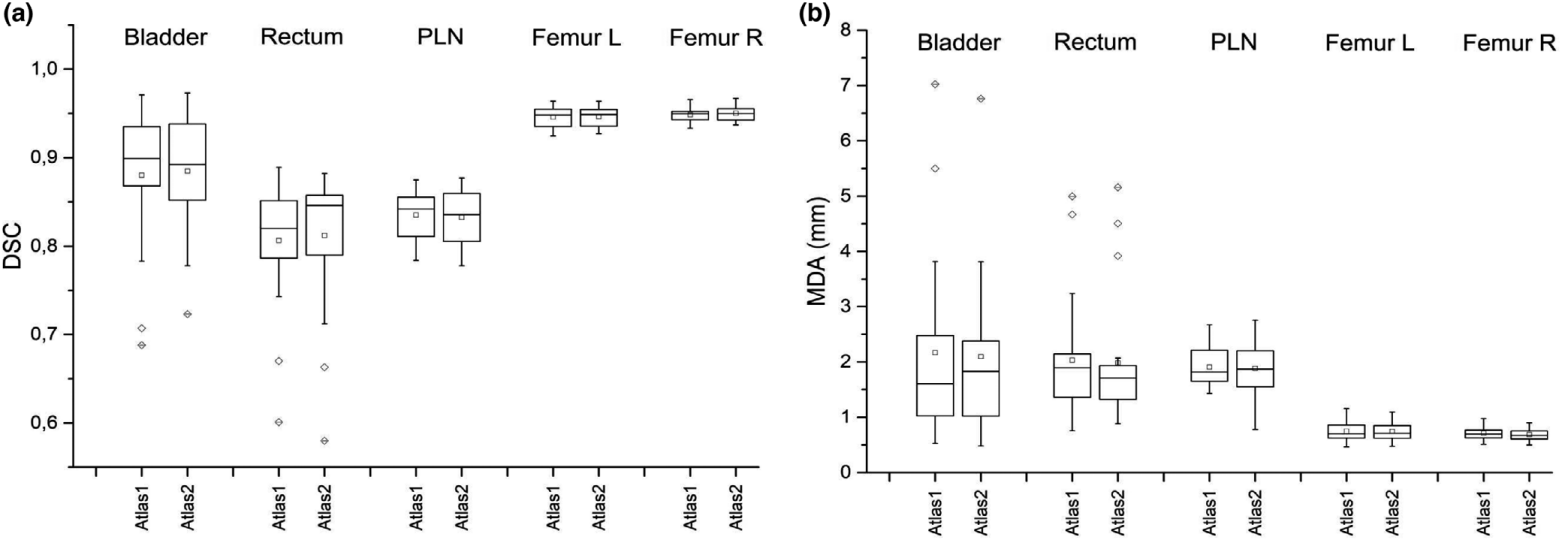
Box plots of dice similarity coefficient (a) and mean distance to agreement (b) between automatic contours, obtained with Atlas 1 and Atlas 2, and reference contours evaluated on a sample of 20 patients for different region of interests.

The comparison between reference and auto‐contours obtained using Atlas 1 and Atlas 2 is reported in Fig. 2, showing DSC and MDA box plot evaluated for each patient and ROI. The statistical tests did not evidence any significant difference between Atlas 1 and Atlas 2 for both DSC and MDA (*P* ≥ 0.2 for all ROIs). This result demonstrates that the registration approach used to register the atlas’ subjects to the template did not influence the accuracy of auto‐contours. Atlas 2 was embedded in the customized MIM auto‐contouring workflow.

#### 3.A.2 Auto‐contouring time

The mean automatic contouring time for Atlas 2 + CT and for Atlas 3 + CT was 27 min while it was reduced to only 9 min for both Atlas 2 + CT crop and Atlas 3 + CT crop. Notably, the FOV crop of the subject to be contoured is effective in reducing the auto‐contouring time, while the FOV size of the atlas subjects is ineffective.

### 3.B Workflow customization and optimization

#### 3.B.1 Deformable image registration algorithm

**Fig. 3:**
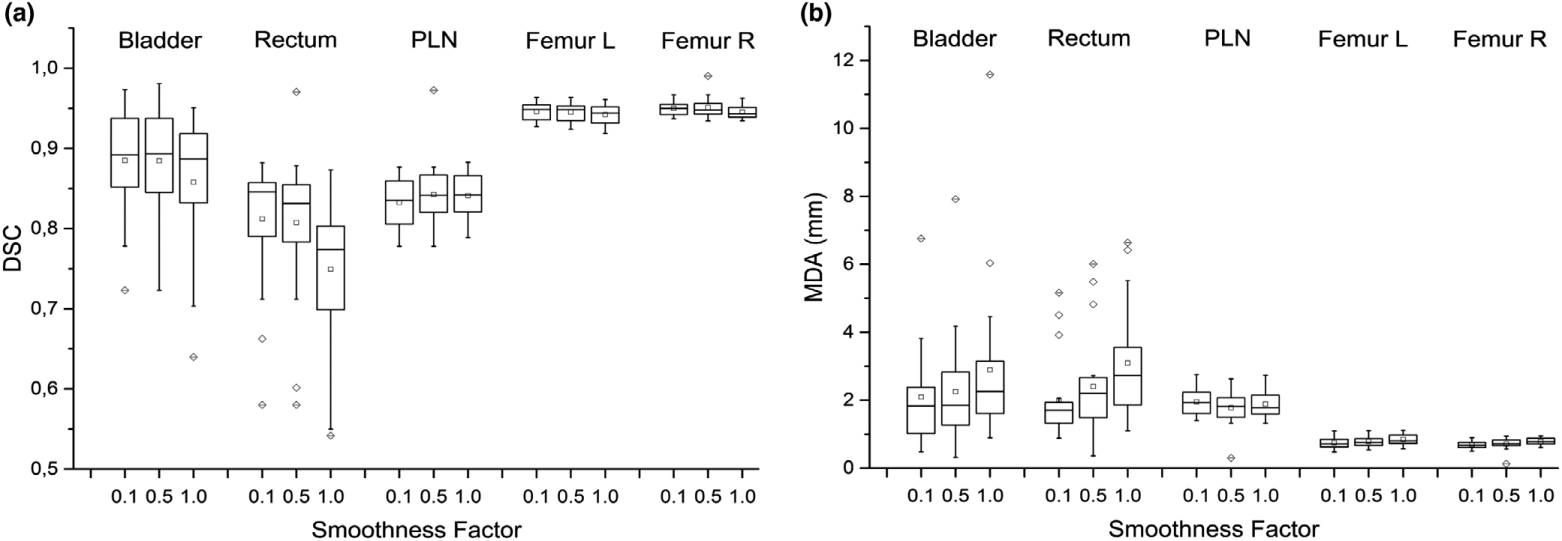
Box plots of dice similarity coefficient (a) and mean distance to agreement (b) between reference contours and automatic contours obtained with *Same‐subject* algorithm varying the smoothing factor and evaluated on a sample of 20 patients.

DSC and MDA similarity indices are reported in box plots of Fig. 3 for each ROI and for three smoothing factors for the Same‐subject algorithm.

The Friedman test for the differences between the three groups showed a statistically significant difference, both for DSC and MDA, for all ROIs except for PLN. The worst results were obtained for SF = 1 where the degree of deformation applied is limited. For DSC, SF = 0.1 resulted significantly better than SF = 0.5 only for left femurs, while for MDA, SF = 0.1 resulted significantly better for all ROIs except for PLN. No difference in contouring time for different SF values was observed. SF = 0.1 was set in our workflow.

**Fig. 4:**
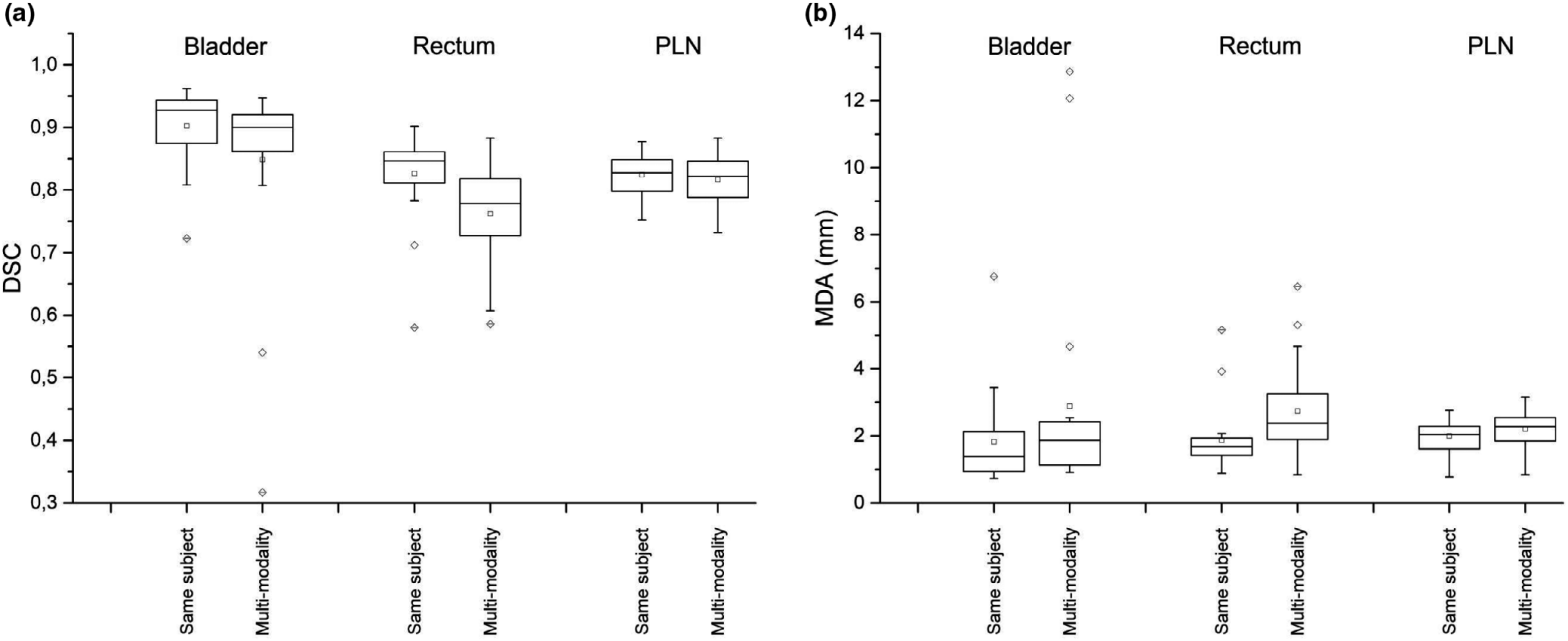
Box plots of dice similarity coefficient (a) and mean distance to agreement (b) between reference contours and automatic contours obtained with *Same‐subject* and *Multi‐modality* on a sample of 20 patients.

The comparison between *Multi‐modality and Same‐subject* algorithms was performed with SF = 0.1 (Fig. 4). As femur auto‐contours were not significantly influenced by the registration algorithm, they are not reported in the plot. DSC data showed worse accuracy for bladder and rectum (*P* = 0.03 and *P* < 0.001, respectively) using *Multi‐modality* instead of *Same‐subject* and equivalent performance for pelvic lymph nodes. For MDA, better or equivalent results were obtained using standard algorithm. A significant MDA reduction was observed for rectum and PLN (*P* < 0.001 and *P* = 0.04, respectively). As for bladder, despite a lower MDA median value for the standard algorithm, it was not possible to detect any significant difference (*P* = 0.08). *Same‐subject* algorithm with SF = 0.1 (instead of 0.5 default value) was embedded in the customized MIM auto‐contouring workflow.

##### Automatic reg refine (ARR) option

**Fig. 5:**
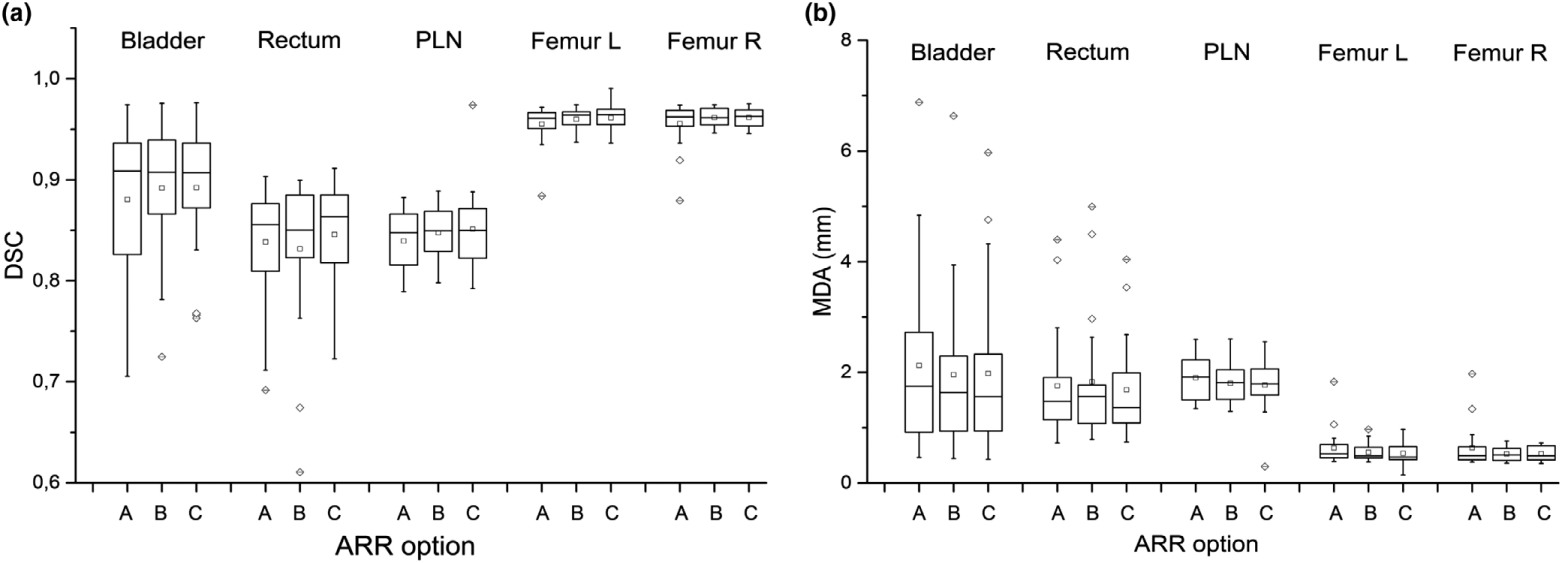
Box plots of dice similarity coefficient (a) and mean distance to agreement (b) for the following settings for the *Automatic Reg Refine (ARR) option*: not applied (a), applied with default settings, that is, 60 mm box spacing and 30 mm box size (b), and applied with customized settings, that is, 30 mm box spacing and 20 mm box size (c).

It was not possible to evidence any significant difference among the three tested options. Both from visual inspection of contours and box plots (Fig. 5), a slight increase in the dispersion of MDA and DSC indices (more evident for bladder, PLN, and femurs) can be noted for contours generated without activating *ARR* option. For this reason, even if the use of *ARR* leads to an increase of computation time, the default option (*ARR* activated with 60 mm box spacing and 30 mm box size settings) was embedded in the customized MIM auto‐contouring workflow.

#### 3.B.2 Finalization algorithm

**Fig. 6:**
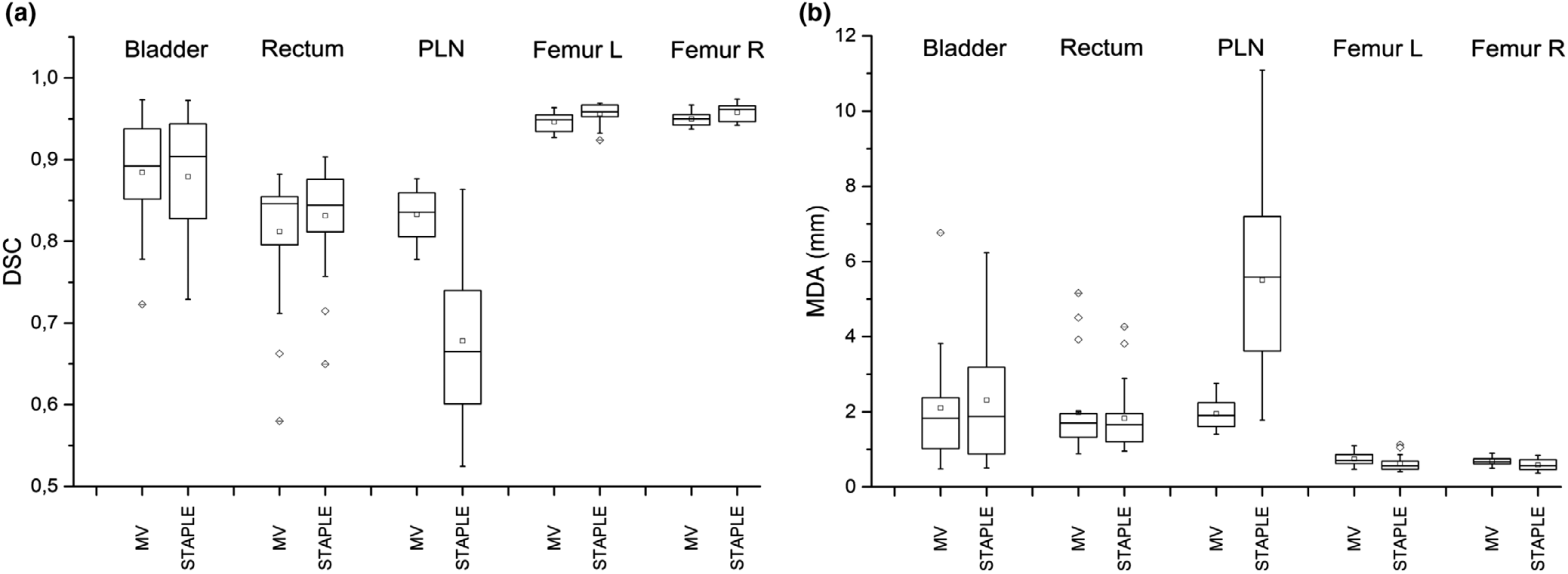
Box plots of dice similarity coefficient (a) and mean distance to agreement (b) between reference contours and automatic contours using MV or STAPLE and evaluated on a sample of 20 patients for different region of interests.

DSC and MDA, calculated for auto‐contours obtained with MV or STAPLE vs reference manual contours, are reported in box plots of Fig. 6.

The bladder auto‐contour was not significantly affected by the finalization algorithm (*P* = 0.6 and *P* = 0.3 for DSC and MDA, respectively). As for rectum, median DSC and MDA were similar: 0.85 and 1.9 mm for MV and 0.84 and 1.7 mm for STAPLE, respectively. Contours obtained with MV or STAPLE were similar even if a statistically significant difference was evidenced for DSC (*P* = 0.03), but not for MDA (*P* = 0.3). The same effect was observed for femurs where the differences between DSC and MDA were statistically significant (*P* ≤ 0.001).

**Fig. 7:**
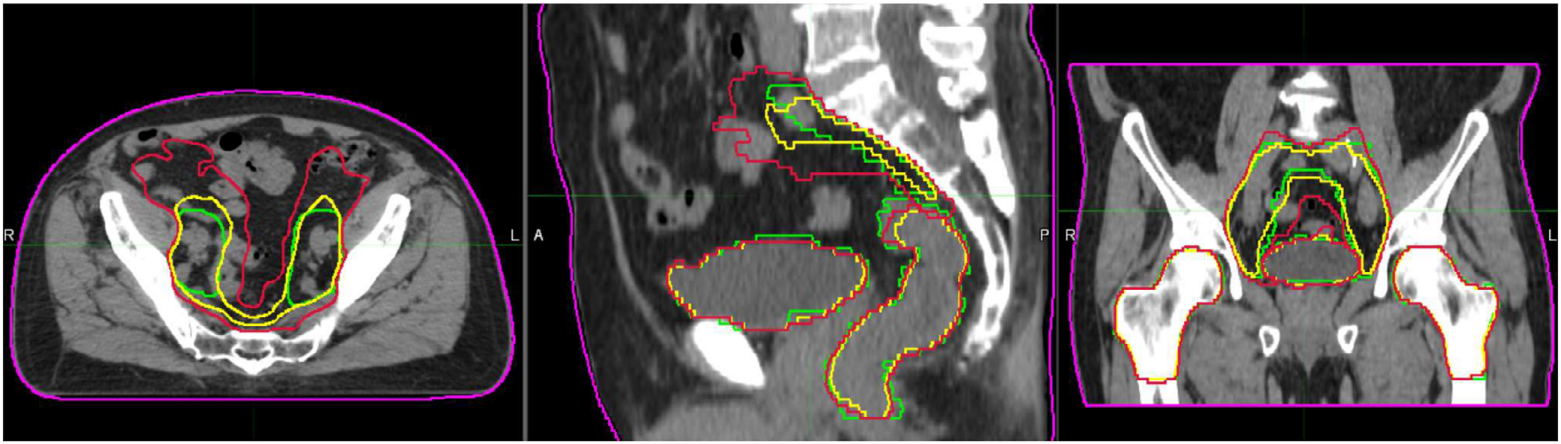
Reference contours (green) and automatic contours extracted with MV (yellow) and STAPLE (red) for one test patient.

On the contrary, PLN auto‐contours obtained with STAPLE showed a clear effect of volume overestimation. Reference contours and automatic contours extracted with MV and STAPLE for one test subject are reported in Fig. 7, as an example. Contours produced with MV resulted evidently more accurate than those produced with STAPLE. This finding was supported by higher DSC and lower MDA for MV (0.83 and 2.0 mm, respectively) than for STAPLE (0.68 and 5.5 mm, respectively). In conclusion, MV was chosen for the definitive workflow, for its strong superiority regarding PLN accuracy.

##### Finalization algorithm: # of k best matching subjects

**Fig. 8:**
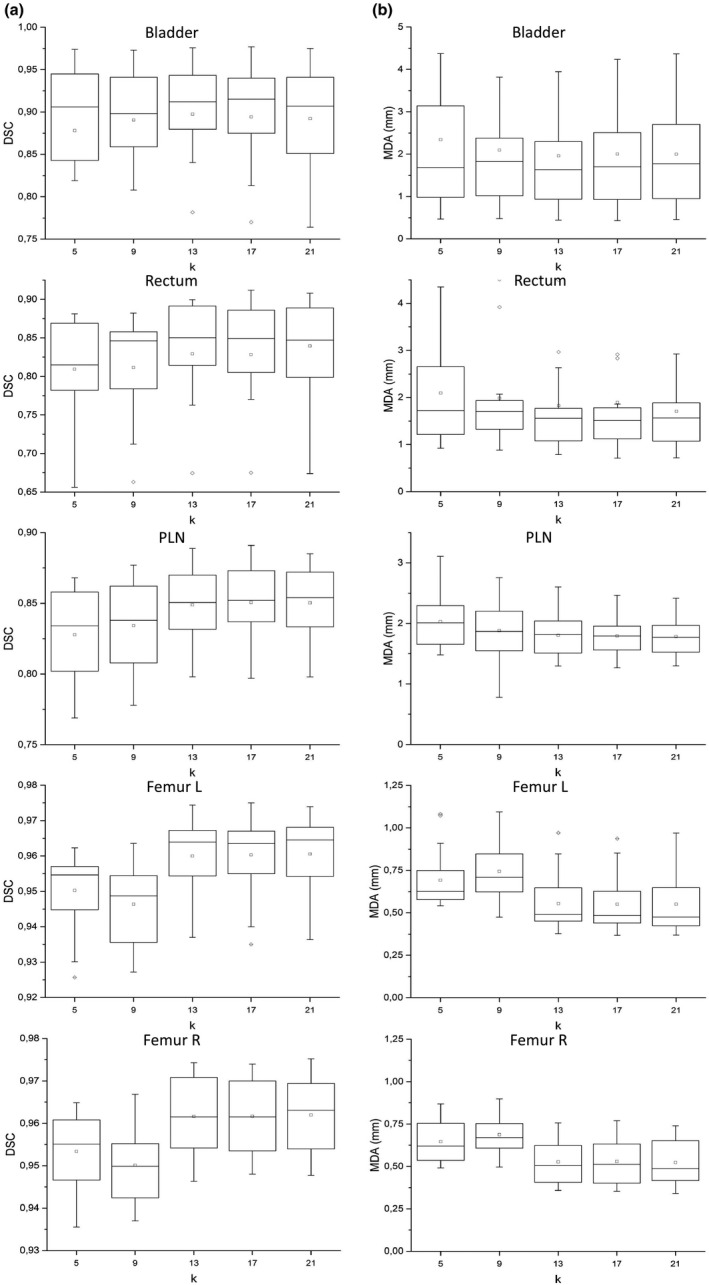
Box plot of dice similarity coefficient (on the left, column A) and mean distance to agreement (on the right, column B) between reference and automatic contours for 20 test subjects varying the number k of best matching subjects.

DSC and MDA values between reference contours and auto‐contours obtained using different k are reported in box plots of Fig. 8 for all ROIs. Contours generated by selecting only five best matching subjects (default option in MIM’s workflow) resulted inaccurate for all ROIs. A significant difference between different k values was observed (*P* < 0.001), for both DSC and MDA, for all ROIs except for bladder (*P* = 0.13 and *P* = 0.08 for DSC and MDA, respectively). For bladder and rectum, the best compromise was obtained for k = 13 while for PLN, the accuracy of contours increases with k, even if the gain is low for k ≥ 13. An increase in contours’ accuracy with higher k value with only a modest DSC gain for k ≥ 13 was also observed for both femurs.

The statistical analysis for differences, limited to k = 13, 17, 21, highlighted no significant difference for any ROI neither for DSC nor MDA. Based on this analysis and considering that contouring time increases approximately linearly with k, k = 13 was embedded in the customized MIM auto‐contouring workflow.

### 3.C Performances of customized atlas and workflow and MIM provided atlas and workflow

**Fig. 9:**
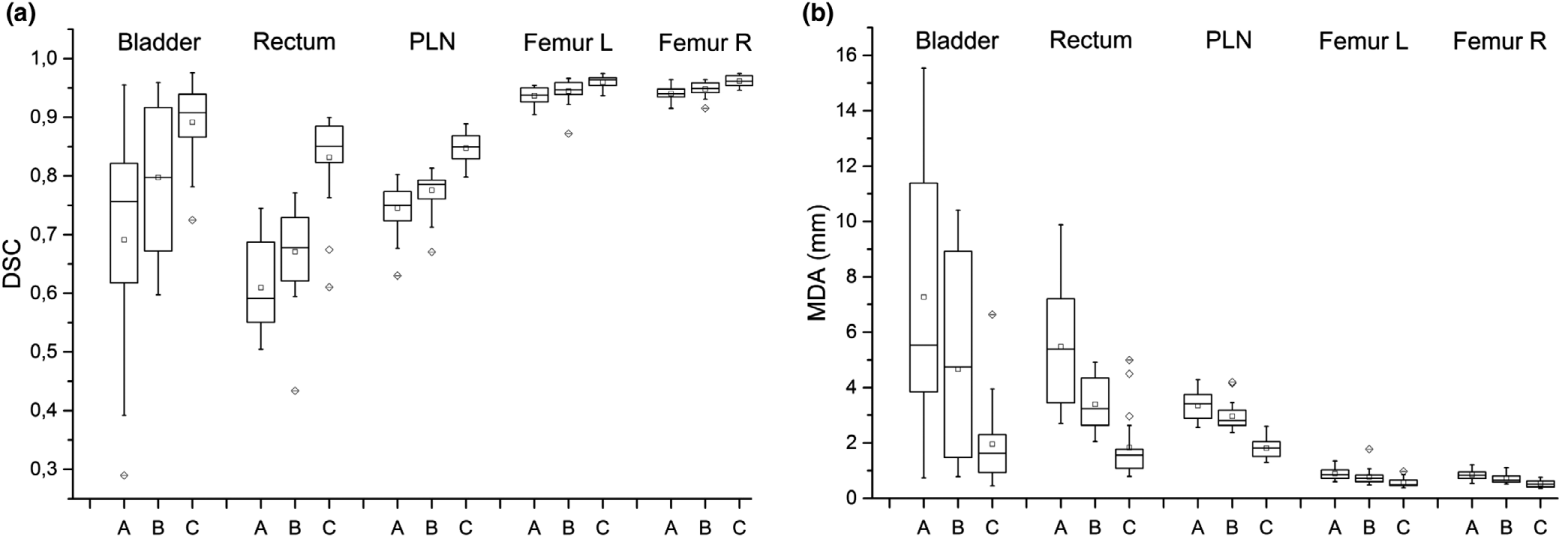
Box plot of dice similarity coefficient (a) and mean distance to agreement (b) between reference contours and automatic contours obtained using (a) MIM Atlas and workflow, (b) MIM Atlas and (c) our customized workflow and our Atlas and customized workflow on the sample of 20 patients for different region of interests.

Figure 9 shows DSC and MDA between reference contours and automatic contours obtained using the following settings: (a) MIM 6.9.5 default atlas and workflow; (b) MIM atlas and our customized workflow; and (c) our atlas and customized.

Both for DSC and MDA, the distribution analysis in terms of position showed that our atlas performs significantly better than MIM default atlas (b–c, same workflow, *P* < 0.005 both for DSC and MDA, all ROIs). Also workflow optimization only, using MIM atlas, leads to a significant improvement of accuracy (A–B *P* < 0.05 for DSC and MDA, all ROIs except Right Femur MDA *P* = 0.06).

Combining the two contributions (atlas and workflow, a–c), median DSC increases from 0.76 to 0.91 for bladder, from 0.59 to 0.85 for rectum, from 0.75 to 0.85 for PLN, from 0.94 to 0.96 for both femurs. Our atlas also showed a better performance in terms of data dispersion reduction for bladder (inter‐quartile range reduced from 0.19 to 0.06) and rectum (from 0.13 to 0.05).

## 4 DISCUSSION

The creation and customization of an atlas‐based workflow for automated contouring require a careful selection of atlas subjects, an accurate contours revision and a thorough tuning of workflow parameters. This is a very time‐consuming task that not all institutions can afford. The aim of this work is to understand how to take full advantage of MIM MAESTRO automatic contouring tools and workflow and to evaluate if it is worth investing time in developing and fine‐tuning a personalized atlas and workflow. This plan has been pursued evaluating the performances of each available tool while developing and optimizing a CT male pelvis atlas based on 55 subjects. The atlas has been optimized on a training sample of 20 subjects randomly extracted from the atlas, with a leave‐one‐out approach.

Regarding the method used to select atlas subjects, various solutions have been proposed and tested in literature: selection based on predefined keywords corresponding to anatomical characteristics; manual selection performed by the operator before each segmentation; selection based on predefined similarity indices (this is the case for MIM MAESTRO). The study of Schipaanboord et al.^39^ suggests that “atlas‐based segmentation with currently available selection methods compares poorly to the potential best performance, hampering the clinical utility of atlas‐based segmentation. Effective atlas selection remains an open challenge in atlas‐based segmentation for radiotherapy planning.” In some studies, different atlases (Small, Medium, Large) based on different patient size and/or anatomical characteristics of the structures to be contoured have been used demonstrating good performances for breast and anorectal cancer.^40^ In this work, we opted for the more general approach including in the same atlas 55 subjects that presented a wide range of anatomical characteristics or size. To choose atlas template, we looked for the specific subject with characteristics common to most subjects. We demonstrated that the strategy used to register each atlas subject on the atlas template did not impact on the created contours accuracy. We did not investigate the effect of using a different template because the definition of a new template necessarily implies a new subjects’ registration on the template, thus introducing a second variable in the comparison. Since we have now demonstrated that the manual subjects’ registration on the template is not critical, it could be possible to further investigate and quantify the choice impact of the template on automatic contours’ accuracy.

Up to 75% time saving, on average, was observed reducing the FOV of the subject to be contoured and, as the time to perform manual FOV reduction is only about 1 min, it is worth to spend time for this preliminary operation.

The DIR algorithms that can be used in the auto‐contouring workflow demonstrated different performances. The default algorithm proposed by the vendor (*Same‐subject*), used with an optimized smoothing factor 0.1 (k = 5 is the default value), has been confirmed to be the most effective compromise between accuracy and calculation time. We demonstrated that better results for pelvic nodes and comparable results for the other structures were obtained using MV instead of STAPLE finalization method. This finding is supported by the study of Acosta et Al.^27^ whose results suggested that the vote decision rule is more robust when applied to a region with high anatomical variability (marked pelvis feature). Also, Wong et al.^24^ found that MV performed better in bladder and prostate, which are characterized by a high anatomical variability.

As for number k choice of best matching subjects, it is very hard to choose the best k on the sole basis of visual examination. Using MV, Acosta et al.^27^ found a DSC saturation with increasing k, at expense of computation time. With STAPLE, they observed that the accuracy had a less stable trend, even decreasing for higher k for some organs. In our study, from a visual inspection of automatically generated contours, only contours generated with k = 5 (default value) seemed to be characterized by an evidently poorer accuracy. Our systematic analysis of accuracy trend as a function of k (Fig. 8) shows that, considering all ROIs, the best compromise between accuracy and calculating time is obtained for k = 13.

Wong et al.^24^ tested k effect with different atlas library sizes (number of subjects composing the atlas varied among 1, 10, 30, 50) and they found that STAPLE assured a better independence respect to the library size. Thus, their final choice was library size = 10 and k = 5, with STAPLE finalization algorithm.

It is worth to be noticed that the trend of contours accuracy in function of k is characteristic of each atlas, probably depending mainly on the atlas size and on the selection method of the best matching subjects. Also, Schipaanboord et al.^39^ recommend that each institution should optimize k depending on the anatomical district and on the particular atlas.

It would also be interesting to evaluate the approach efficacy of the best matching subjects’ selection adopted by the software MIM MAESTRO. We have ascertained that the selection of k best matching subjects, for each test patient, is influenced by atlas template choice. Furthermore, changing the test subject among the 20 test subjects (randomly sampled from the 55 atlas subjects), the variability of the atlas subjects chosen as best matching subjects is limited, suggesting that there is a stable group of atlas subjects, common for all the 20 test subjects.

To evaluate whether it is worth to invest time in atlas and workflow creation and customization, we compared the performance of MIM Atlas and workflow; MIM Atlas and our customized workflow; and our Atlas and customized workflow.

It is a must to point out that both reference contours and atlas subjects have been contoured and reviewed following criteria and guidelines adopted by our institution. On the contrary, MIM atlas subjects were contoured by other physicians, in a different institution. This could introduce a bias in favor of our atlas.^12^ Indeed, similarity indices, used in this study for testing statistical difference between the three combinations, are referred in all cases to a pool of test patients which were reviewed for consistency by a radiation oncologist of our institution. For this reason, the test results only establish whether there is a statistically significant difference between these three groups of similarity indices and needs a proper interpretation.

**Fig. 10:**
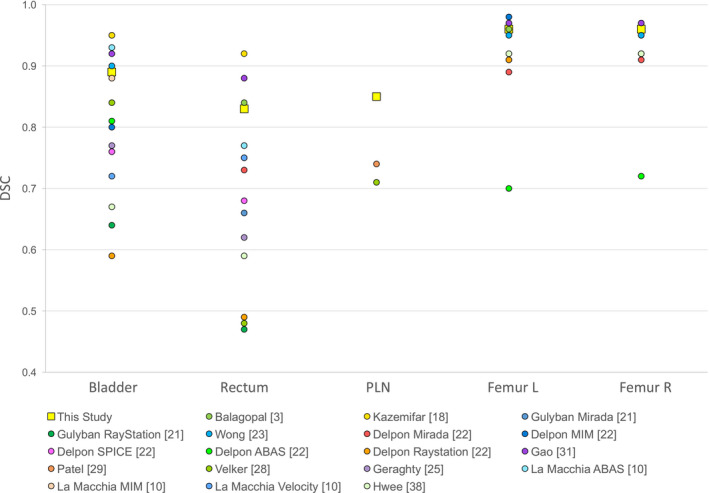
Mean dice similarity coefficient comparison of automatic contours of various region of interests (Bladder, Rectum, PLN, Femur L, and Femur R) obtained by different authors. The results obtained in the present study are depicted with a bigger square. Error bars are not displayed (sigma was on the order of 0.05).

The increase in DSC and decrease in MDA, obtained with our atlas, are due both to contouring criteria standardization and to atlas construction optimization. We suggest that each institution creates and optimizes its own atlas, based on a sample of subjects extracted from local population and contoured according to standardized criteria. Finally, due to the arbitrariness of reference contours, DSC cannot be used as an absolute measurement of accuracy. DSC should only be used to compare contours obtained with different methods and all referred to the same reference contours. Nevertheless, all studies about atlas optimization report mean DSC values of the obtained automatic contours. In Fig. 10, our results are approximatively compared to those found in literature.^3^, ^32^, ^41^ Our results are satisfactory when compared to many atlas‐based studies and are comparable to those based on deep learning.^3^, ^19^ Of note, few studies in literature include PLN in the atlas and this is an added value of our study.

## 5 CONCLUSION

The results of automated contouring are highly dependent on criteria standardization and contours accuracy of the atlas subjects. We suggest a thorough optimization of the atlas‐based segmentation tool, compatibly with data availability, radiation oncologists’ expertise, and time to spend.

In the case of MIM MAESTRO software, the deformable registration algorithm shows high performances and the default options are already tuned by the vendor and we only modified the smoothing factor for deformable registration.

We suggest focusing atlas optimization and workflow mostly on the template choice, the optimal number of best matching subjects, the postprocessing options, the FOV optimization (eventual reduction) of the subject to be contoured. Particularly, the FOV reduction of the subject to be contoured has proven to be the most effective way to reduce the time necessary for automatic contouring (up to 75% time saving: on average from 27 to 9 min).

## AUTHORS' CONTRIBUTIONS

S. Pallotta devised the project; S. Pallotta, M. Casati, and S. Piffer designed the study; G. Simontacchi, V. Di Cataldo, D. Greto, I. Desideri, M. Vernaleone, G. Francolini, and L. Livi selected the subjects; G. Simontacchi contoured the atlas subjects; S. Piffer and M. Casati worked on atlas creation and optimization, tested workflow options, elaborated the data and analyzed them, with the contribution of S. Calusi and L. Marrazzo; M. Casati drafted the manuscript with the support of S. Pallotta and S. Piffer; S. Pallotta aided in interpreting the results and worked on the manuscript; All authors discussed the results and commented on the manuscript.

## Supporting information


**Table S1**. Commercial solutions for automated atlas‐based segmentation.
