# Supplementary material for: Methodological approach to create an atlas using a commercial auto‐contouring software
Source: J Appl Clin Med Phys. 2020 Nov 25;21(12):219–30. doi: 10.1002/acm2.13093 (PMC7769405; doi:10.1002/acm2.13093)
Supplement: Supplementary file 1 — Table S1. Commercial solutions for automated atlas‐based segmentation. [file ACM2-21-219-s001.docx]

**Table S1.** Commercial solutions for automated atlas-based segmentation

| Model Based | Atlas Based | Deep Learning | Vendor | Software | TPS  integration |
| --- | --- | --- | --- | --- | --- |
|  | x |  | Mirada Medical L.t.d. | WorkFlow Box | no |
|  |  | x | Mirada Medical L.t.d. | ‘‘Mirada DLC Expert” prototype | no |
|  | x |  | MIM Software Inc. | MIM Maestro | no |
| x | x |  | Philips | SPICE | yes |
| x | x |  | Elekta | ABAS | no |
| x | x |  | RaySearch Laboratories | RayStation | yes |
|  | x |  | VELOCITY VARIAN | Velocity AI | No |
|  | x |  | VARIAN | Smart Segmentation Knowledge Based Contouring | yes |
| x | x |  | Accuray | Multiplan Autosegmentation | yes |
|  | x |  | Dosisoft | IMAgo | yes |
|  | x |  | BRAINLAB | iPlan CMF planning software | yes |
